# Environmental quality outlook of the leading oil producers and urbanized African states

**DOI:** 10.1007/s11356-023-28915-w

**Published:** 2023-08-22

**Authors:** Stephen Taiwo ONIFADE, Andrew Adewale ALOLA

**Affiliations:** 1 School of Finance and Accounting, University of Vaasa, 65200 Vaasa, Finland; 2 Faculty of Economics, Administrative, and Social Sciences, KTO Karatay University, Konya, Turkey; 3 CREDS-Centre for Research On Digitalization and Sustainability, Inland Norway University of Applied Sciences, 2418 Elverum, Norway; 4 Faculty of Economics, Administrative, and Social Sciences, Nisantasi University, Istanbul, Turkey; 5 Adnan Kassar School of Business, Lebanese American University, Beirut, Lebanon

**Keywords:** Environmental quality, Income, Urbanization, EKC, Africa

## Abstract

This study seeks to explore the links between energy consumption and environmental quality in the wake of rapid urbanization in Africa with empirical insights from the cases of Libya, Morocco, Nigeria, Algeria, Angola, Egypt, and South Africa. These countries aside from being among the largest economies; are also among the leading energy producers and the most urbanized economies that emit the most carbon dioxide on the continent. Based on the Pooled Mean Group (PMG) panel ARDL estimator, the dynamics nexus between the variables was estimated vis-à-vis the short-run and long-run coefficients using relevant sample data between 1990 and 2015. The study further examines the channels of causality between the variables while also testing for the validity of the popular Environmental Kuznets curve (EKC) hypothesis for the panel of countries. The results confirm that the rising level of energy use significantly exacerbates the level of carbon emission among the countries in the study while growing urbanization significantly creates a negative impact on carbon emission. In addition, an increase in per capita income improves the environmental quality but the doubling of income per capita triggers environmental degradation, thus invalidating the EKC hypothesis in the examined panel economies. In essence, these countries have not reached the supposed turning point at which income growth can yield desirable emission mitigation effects. Following the findings, essential recommendations are provided for policymakers in the main text.

## Introduction

The global concerns surrounding the adverse impacts of rising carbon dioxide (CO_2_) emissions amidst the quest to sustain incremental economic growth by many nations have cut the attention of more policymakers and researchers in recent times. These emissions constitute a significant proportion of the global Greenhouse Gas (GHG) emissions. According to Olivier and Peters (2019), a 2.0% increase in the global GHG in the year 2018 was mainly due to the 2% rise in global CO_2_ emission that was experienced in the same year. The rising CO_2_ emission combined with other greenhouse gases like CH_4_, N_2_O, and F-gases among others constitute the major GHGs that consistently worsen the global climate change challenge in the last decades. The Intergovernmental Panel on Climate Change (IPCC 2018) noted that global climate change especially in the twenty-first century has remained a dire challenge and threat to human existence just as the impacts of these changes are currently being felt across the globe although with varying degrees of severity from one nation to another (IPCC 2018).

Over the years, incremental economic growth has also been closely associated with an increase in energy consumption alongside the rise in the rate of urbanization that is often observed as nations become industrialized (Jones 1991; Alola et al. 2020; Rafindadi and Usman 2021; Onifade et al. 2023a). However, in most cases, this development also paves the way for the rise in CO_2_ emissions due to the higher energy demand especially from unclean energy sources such as coal and fossil fuels energy utilization (Harmsen and Graus 2013; Salahuddin et al. 2018; Alola and Alola 2018; Alola et al. 2019a; Bekun et al. 2019). Similarly, evidence from previous studies has equally linked environmental challenges with the trend of economic integration and globalization especially because of the dependence of many states on energy utilization (Alola and Joshua 2020; Bekun et al. 2020; Joshua et al. 2020; Saint Akadiri et al. 2019, 2020a, b).

In 2018, global consumption of oil products increases by 1.2% while that of natural gas increased by 5.3%. With this, India, China, and the United States of America (USA) account for a respective increase of about 5.1%, 5.0%, and 2.1% of the global increase in oil product consumption. Concerning the 5.3% global increase in natural gas consumption, China, the USA, and Russia account for about 17.7%, 10.5%, and 5.4% respectively (Olivier and Peters 2019). Importantly, the rise in energy consumption has consistently accounted for the negative consequences of the growing CO_2_ emissions globally (Alola 2019a, b; Alola and Kirikkaleli 2019; Onifade et al. 2023b; Usman et al 2020). With the global trend of increased carbon emissions, the rise in average temperature, extreme climatic changes with likely threats of loss of natural habitats due to cases of severe droughts and dangerous precipitations among other issues as overwhelmingly inevitable (Liu and Bae 2018; Ağbulut et al. 2019; Alola et al 2019b; Ibrahim and Alola 2020). Therefore, the call to reduce global CO_2_ emissions has continued to be on the rise, especially with more attention on developed and emerging economies.

Currently, more nations are consciously pushing for development and economic growth with the use of available resources such as substantial deposits of oil, natural gas, and coal reserves at their disposal. However, studies have shown that this growth push is often executed at the expense of the environment (Gyamfi et al. 2023; Bekun 2022). For instance, most of the African states are home to many natural resources in addition to the continent having a huge young population and rapid urbanization (Onifade 2022; Balcilar et al. 2023). Ironically, most of these African states have been noted to be in a disadvantageous situation in the events of growing climate change disasters since they lack proper resilience capacity, mitigations programs, and adaptation infrastructures (Dingru et al. 2023; Onifade 2023; Appiah et al. 2022).

Given this background motivation, this study seeks to explore the causal nexus and long-run association between energy consumption and carbon emissions in the wake of rapid urbanization in Africa. In this context, the current study explores the empirical insights from the case of Algeria, Angola, Egypt, Libya, Morocco, Nigeria, and South Africa. These countries aside from being among the leading oil producers on the continent with huge dependence on fossil fuels for energy generation, these countries are also among the largest economies that are highly urbanized. In addition, the countries are mostly the largest carbon dioxide-emitting countries in the continent. Hence, the selection of the examined countries is expected to give more credence to the recommendations and policy directions for these nations and other African economies based on the overall empirical evidence from the analysis.

To provide an effective readership, the other section of the study is carefully outlined. In “The trend of carbon emission and urbanization in Africa” section, the trend of carbon emissions amidst urbanization is carefully outlined. Subsequently, relevant literature that supports the contextual framework of the study is detailed in “Literature review and the underpinning” section. In “Data and methodology”, “Results and discussion”, and “Conclusion and policy recommendations” sections, we offer the data description with methodology, discussion of the results, and the conclusion respectively.

## The trend of carbon emission and urbanization in Africa

Rising urbanization and its attendant challenges are not new issues in the world at large but the recent dynamics of the rate of urbanization in Africa have continued to attract the attention of policymakers and governments on the continent and beyond (Erdoğan et al. 2022). As of 2019, Africa has become the home to approximately 1.36 billion people with over 80% of this population found in the Sub-Saharan Africa (SSA) region (WDI 2019). In the SSA region, the urban population as a percent of the total population has risen to 40.71% in 2019 compared to the 14.69% that it used to be in 1960 (WDI 2019). Based on the information from OECD/SWAC (2020), Africa is the continent with the highest urban growth rate, and the current population is expected to double over thirty years between 2020 and 2050. Of course, there are pros and cons to this development. On the pros side, for instance, rapid urbanization has the potential of transforming a city into a major hub for economic and financial activities among other positive sides of urbanization (Ravallion 2008; Nguyen and Nguyen 2018). On the other hand, the socioeconomic effects of worsening urban poverty rates, unemployment, and an increase in the proportion of the population without access to proper sanitation facilities as the number of people living in slums grows may pose more threats to the possible benefits of urbanization. Besides, there could also be an environmental cost of rising urbanization as pressure mounts on social amenities while the demand for energy consumption rises. The nexus between this development vis-à-vis the challenges of rising CO_2_ emissions among other environmental pollutants have been explored in empirical studies (Asongu et al. 2020; Usman 2023; Taiwo et al. 2022; Xie et al. 2020).

**Fig. 1:**
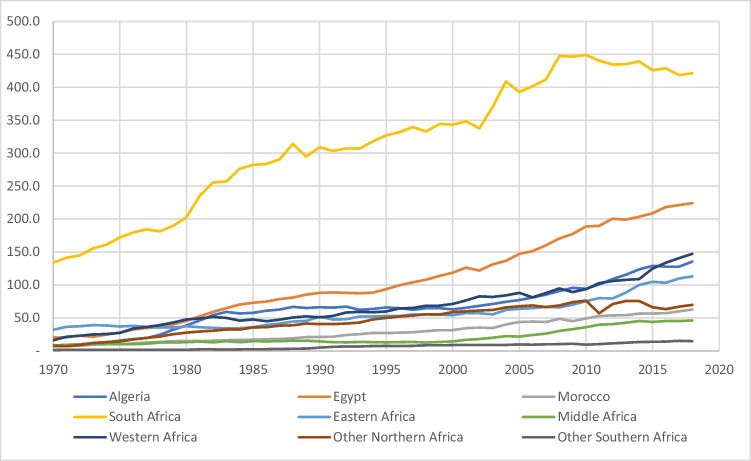
CO_2_ emission in million tonnes in Africa 1970–2018. Source: Data from BP Statistical Review of World Energy (2019)

Therefore, the call to reduce global CO_2_ emission has consistently been on the rise and with more attention on the developed and emerging economies as countries in this economic subdivision contribute the highest amount of CO_2_ emissions on the global level (Onifade and Alola 2022; Onifade et al. 2022). As of 2018, the most carbon dioxide emissions are from Asia Pacific countries (49.41%), North America (18.2%), Europe (12.5%), the Middle East (6.3%), the Commonwealth of Independent States, CIS (6.2%), South and Central America (3.8%), and Africa (3.6%) respectively (BP 2019). Although Africa’s contribution to global carbon emission is relatively low compared to other continents, however, CO_2_ emission in the continent has also experienced a steady rise over the years (see Fig. 1). In specific, Africa experienced a rise in carbon emission from an average of about 211.1 million tonnes of CO_2_ emitted in the late 1960s to an average of 1150.4 million tonnes of CO_2_ emission between 2010 and 2018 representing an approximate 544.9% increase within the period (BP 2019).

**Fig. 2:**
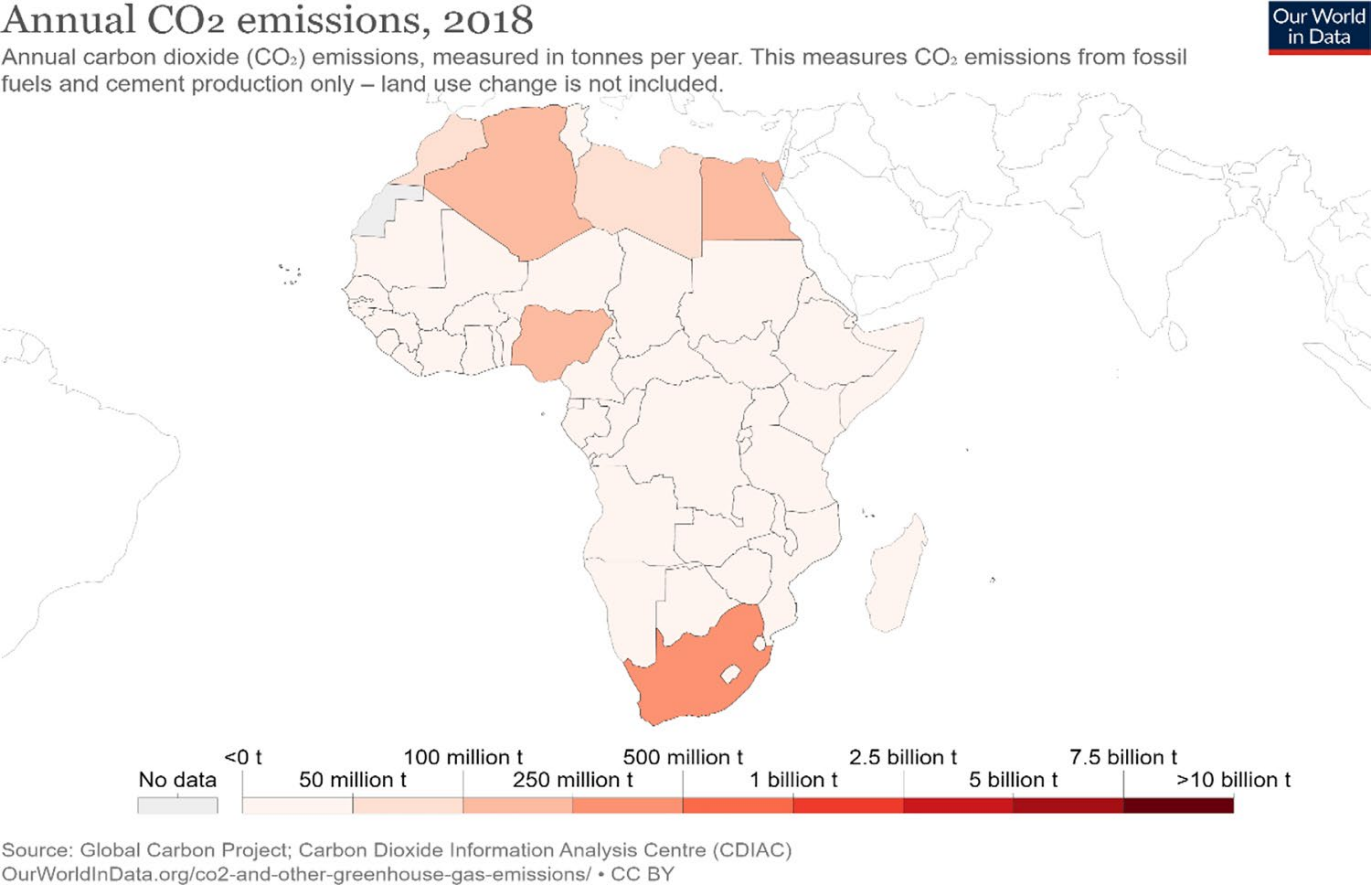
Annual CO_2_ Emissions in Africa (2018)

The annual CO_2_ in Africa as of 2018 is shown in Fig. 2, and countries like Algeria, Egypt, Nigeria, Libya, Morocco, and South Africa appear to be emitting more CO_2_ on the continent. Oil and gas play an important role in the global economy and it is the backbone of some of these African economies due to the substantial amount of fossil fuel reserves. At the end of 2019, about 8.15% of the world’s total proven oil reserves are in Africa out of which Libya, Nigeria, Algeria, Angola, and Egypt account for more than 87.52% of the entire proven oil reserves on the continent (OPEC 2020). Contrastingly, in 2018, the combined amount of primary energy consumption fueled by oil and natural gas as a percentage of the total primary energy consumption in Africa stood at 69.4%, with other energy sources like coal, nuclear energy, hydroelectric, and renewables accounting for 21.8%, 0.54%, 6.52%, and 1.56% respectively (BP 2019). Invariably, the total amount of primary energy consumption fueled by nonrenewable sources including oil, gas, and coal accounts for 91.2% of the total energy consumption on the continent. Hence, there is an urgent need to redress the present status quo as far as environmental sustainability is concerned in Africa in the wake of growing energy demand and the urbanization dynamics in recent times.

## Literature review and the underpinning

The attention of the global community has been drawn to the rising trend of environmental degradation created by unclean energy consumption and its possible consequence on our planet at large. Thus, studies on the dynamic nexus between economic growth and energy consumption vis-à-vis the amount of environmental pollutants emissions have become more appealing to researchers lately. In this regard, the Environmental Kuznets Curve (EKC) has played a huge role in providing strong theoretical underpinning in the empirical literature based on the original work of Kuznets (1955). The EKC holds that the economic growth and environmental degradation nexus follows an inverted U-shape curve such that the adverse impacts of growth vis-à-vis the amount of degradation on the environment will become reduced or offset at a certain level of economic growth even though such adverse effects are pronounced at an initial stage (Selden and Song 1995; Usman et al. 2021). The EKC has been applied to explore the environmental pollutants-growth dynamics of many economies and the empirical evidence has shown mixed results (Shahbaz et al. 2013; Sarkodie and Strezov 2018; Appiah et al. 2023; Adedoyin et al. 2020).

Furthermore, the mixed findings that characterized the empirical literature over the years have led to the existence of varying degrees of causality between economic growth indicators and environmental pollutants like CO2 emissions. Some studies have suggested a unidirectional causality between economic growth and energy consumption such as the study of Soytas and Sari (2003) which found evidence of unidirectional causality from energy consumption to economic growth in four economies including Turkey, France, West Germany, and Japan. Additionally, Yang and Zhao (2014), and Sa'ad (2010) also obtained unidirectional causality between energy consumption and growth for the cases of India and Nigeria respectively. On the other hand, some other studies have also suggested a bidirectional causality on the contrary. For example, the study of Wang et al., (2011) and that of Liu and Bae (2018) have come up with findings that support a bidirectional causality between emission and industrialization that has induced growth in the case of China especially when considering the long-run analysis.

Moreover, the study of Ilesanmi and Tewari, (2017), and Mirza and Kanwal (2017) have provided similar evidence in support of the existence of a bidirectional causality in the energy-growth nexus for the case of South Africa and Pakistan respectively. Soytas and Sari (2003) also found a bidirectional causality in the case of Argentina among some other developed and emerging economies. Although unidirectional and bidirectional causality has dominated the literature, it is, however, intriguing to also note that there are empirical findings that support no causal relationship between economic growth and energy consumption indicators. For instance, the study of Soares et al. (2014) concluded that there was no evidence in support of long-run causality between growth and energy consumption in the case of Indonesia.

In general, most recent studies have shown the exacerbating effects of economic growth and energy indicators on environmental degradation rates and some of these studies have also been extended to cover African nations. However, most of these studies have looked at the African context as a single bloc, thereby neglecting the importance of region-specific or country-specific characteristics as the case may be. Usman et al. (2022) noted that growth as seen in income levels significantly increases carbon emissions in Africa. In another study, Djellouli et al. (2022) discovered that non-renewable energy use generally creates a positive effect on emissions in Africa while renewable use shows a negative effect on pollution from carbon emissions on the continent. They also went further to reveal that the environmental degradation in Africa has been positively induced by economic growth while noting that the EKC does not hold for the continent. Thus, the findings of (Djellouli et al. 2022) partly converge with the submissions of Usman et al. (2022) as far as the environmental effect of growth is concerned but differ on the aspect of the EKC given that the latter omits the EKC examination.

In a nutshell, the various conclusions from the empirical literature indicate that the results might have been influenced by the country’s specific differences and methods of analysis. It has been noted that there is a need to give attention to country-specific differences in a study to arrive at insightful policy recommendations on relationships among interacting variables which in this case, the nexus between energy consumption and economic growth is of interest to us (Akpan and Akpan 2012). Hence, this study seeks to explore the links between economic growth and energy consumption using newer approaches for country-specific scenarios of the leading oil producers and urbanized African states. The merits of this kind of study should not be sidelined considering the apprehensions surrounding the possible consequences of rising CO_2_ emissions amidst the quest for sustainable economic growth in recent times.

## Data and methodology

All data measurement was taken in natural logarithms for the variables across the seven African countries (Algeria, Angola, Egypt, Libya, Morocco, Nigeria, and South Africa) in the study. Data were obtained from the World Bank development indicator (WDI 2019). The natural log of carbon dioxide (CO_2_) emissions in metric tons from each country between the period of 1990 and 2015 captures the level of environmental pollution. Following Sadorsky (2014), the natural logarithm of the proportion of the population living in urban settlements was used as a proxy for urbanization (URB). Economic growth (PCI) was captured by the real GDP per capita of each nation in constant 2010 US$. The amount of energy consumption (EGC) was represented in the model by taking the natural log of energy use in kilograms of oil equivalent per capita for each country. In addition, the square values of the real GDP per capita (PCI^2^) were introduced into the model to check if the Environmental Kuznets Curve hypothesis holds among these countries (Sadorsky 2014; Salahuddin et al. 2018). The statistical properties which specifically illustrate the mean, and deviation from the mean (including the maximum and minimum values) of the explored variables are depicted in Table 1.

**Table 1:** Statistical properties of the variables

	Carbon emissions	Per Capita Income	Urbanization	Energy Consumption	No. of observation
ALGERİA					
Mean	0.4982	1.5010	1.7888	2.9884	26
Maximum	0.5860	2.2523	1.8503	3.1230	
Minimum	0.4278	1.1696	1.7167	2.9042	
Std. Dev	0.0417	0.2871	0.0413	0.0670	
ANGOLA					
Mean	-0.1243	3.2380	1.7072	2.6786	26
Maximum	0.2213	10.5988	1.7974	2.7422	
Minimum	-0.5404	0.6370	1.5699	2.6371	
Std. Dev	0.2129	3.3356	0.0692	0.0318	
EGYPT					
Mean	0.2904	1.9220	1.6330	2.8489	26
Maximum	0.4103	2.2586	1.6383	2.9598	
Minimum	0.1289	1.5479	1.6300	2.7329	
Std. Dev	0.1003	0.1809	0.0020	0.0847	
LIBYA					
Mean	0.9414	1.8295	1.8865	3.4547	26
Maximum	0.9999	2.71097	1.8991	3.5255	
Minimum	0.8031	1.3730	1.8791	3.3406	
Std. Dev	0.0355	0.2152	0.0062	0.0423	
MOROCCO					
Mean	0.1076	1.4377	1.7264	2.6384	26
Maximum	0.2517	2.8222	1.7840	2.8436	
Minimum	-0.3854	1.2883	1.4725	2.4873	
Std. Dev	0.1358	0.2813	0.0577	0.0963	
NIGERIA					
Mean	-0.2634	1.7400	2.8637	1.5717	26
Maximum	-0.0921	2.8222	2.9023	1.6798	
Minimum	-0.5058	1.2740	2.8332	1.4725	
Std. Dev	0.1445	0.4642	0.0200	0.0654	
**SOUTH AFRICA**					
Mean	0.9416	2.0594	1.7648	3.4105	26
Maximum	0.9991	2.5197	1.8118	3.4698	
Minimum	0.8880	1.7572	1.7163	3.3600	
Std. Dev	0.0280	0.2077	0.0293	0.0266	

Std. Dev is the Standard Deviation

### Models and estimation procedures

To explore the nexus among the variables, we set up the multivariate algebraic expression of the relationship among the variables as shown in Eq. (1);

1 $$Log\left({C{O}_{2}}_{it}\right)={\alpha }_{0}+{\alpha }_{1}Log{URB}_{it}+{\alpha }_{2}Log{EGC}_{it}+{\alpha }_{3}Log{PCI}_{it}+{\alpha }_{4}Log{{PCI}^{2}}_{it}+{\varepsilon }_{it}$$

Here, the observations are provided on the variables for each country *i* at a given time *t* and ${\alpha }_{0}$ is the intercept parameters and ${\alpha }_{1},{\alpha }_{2},{\alpha }_{3},$ and ${\alpha }_{4}$ are the slope parameters of the model respectively. The conversion of the variables in natural logarithms helps to ensure interpretations as elasticities. All the variables assume their previous definitions as provided in “Models and estimation procedures” section. We proceeded to examine the unit root (see Table 2) properties of each variable as obtainable in existing literature (Salahuddin et al. 2018; Taiwo et al. 2020; Adedoyin et al. 2020; Onifade et al. 2020a).

**Table 2:** Panel Unit Root Results

Statistics	*Test at First Difference (intercept and trend)*				
Variables	LogCO_2_	LogURB	LogEGC	LogPCI	LogPCI^2^
LLC	-6.03983***	-3.28911***	-7.56049***	-6.25640***	-5.28841***
Breitung	-7.26151***	2.05751	-3.86300***	-2.90939**	-2.06220**
IPS	-6.65981***	0.66317	-7.91532***	-7.27887***	-6.81615***
Fisher-ADF	64.9181***	23.2714**	75.7344***	75.7844***	72.0908***
Fisher-PP	611.351***	9.35704	278.096***	81.9253***	73.7229***

The subscripts *** and ** show the statistical significance of estimates at 1% and 5% levels respectively. LLC and IPS are the unit root test for Levin et al. (2002) and Im et al. (2003) respectively. LogCO_2_ is carbon dioxide emissions, LogEGC is the energy use in kilograms of oil equivalent per capita, and LogURB is the proportion of the population living in the urban settlements. LogPCI and LogPCI^2^ are the real GDP per capita and the square values of the real GDP per capita respectively

The unit root test was performed on each variable at the first difference using the Fisher-ADF test (ADF 1979), Phillips and Perron test (PP 1988), Breitung (2000), Levin et al. (2002) test, and Im et al. (2003) unit root test (see Table 2). The test conducted follows the model that included both the intercept and trend parameters. The output of the results provided evidence in support of the stationarity properties of all variables at their first difference. Following contemporary studies (Salahuddin et al. 2018; Yussif et al. 2022; Adedoyin et al. 2020; Onifade et al. 2020b; Çoban et al. 2020) having confirmed the integration order of our variables, we proceeded to test for cointegration relationships between the variables using the Pedroni (1999) and Pedroni (2004) cointegration technique. This approach provides outputs on seven test statistics including the group rho statistics, the group ADF-statistics, the group PP-statistics, the panel v-statistics, the rho statistics, the PP-statistics, and the ADF-statistics. The results of the Pedroni (1999) and Pedroni (2004) cointegration test is provided in Table 3.

**Table 3:** Pedroni panel cointegration test outputs

Alternative hypothesis: common AR coefficients. (Within-Dimension)				
	Stats	Prob	Weighted stats	Prob
Panel v-Statistic	-2.637053	0.9958	-2.183135	0.9855
Panel rho-Statistic	-0.815203	0.2075	-1.525345	0.0636
Panel PP-Statistic	-4.418430	0.0000***	-6.572740	0.0000***
Panel ADF-Statistic	-4.103023	0.0000***	-2.940805	0.0016***
Alternative hypothesis: individual AR coefficients. (Between-Dimension)				
	Statistic	Prob		
Group rho-Statistic	-0.273059	0.3924		
Group PP-Statistic	-5.953347	0.0000***		
Group ADF-Statistic	-2.715970	0.0033***		

The subscripts *** and ** present the significance of estimates at 1% and 5% levels respectively

From the results in Table 3, it is concluded that there is a long-run relationship among the variables following the statistical significance of a combination of about six of the reported test statistics from the Pedroni cointegration technique. Hence, we proceeded to explore the long-run coefficients of the variables by applying the Pooled Mean Group (MPG) autoregressive distributed lag model (ARDL) of Pesaran et al. (1999).

#### The PMG ARDL and Granger causality

Given that the standard ARDL estimation models are not effective at controlling potential bias from the connection between the mean-differenced autonomous factors and the disturbance term, the PMG estimator by Pesaran et al. (1999) is therefore employed. The PMG estimator is robust for model series with a mixture order of integration. Additionally, the ARDL/PMG estimator is also unique because it simultaneously presents a long and short-run dynamic analysis as well as providing a country-specific short-run estimate. Thus, by applying the PMG technique to Eq. (1) above, the following expression is presented:

2 $$\Delta {lny}_{it}={\varnothing }_{i}{ECT}_{it}+\sum\nolimits_{j=0}^{q-1}\Delta {lnX}_{1(t-j)}{\beta }_{ij}+\sum\nolimits_{j=1}^{p-1}{\psi }_{ij}\Delta {lny}_{i(t-j)}+{\varepsilon }_{it}$$

3 $${ECT}_{it}={y}_{i(t-1)}-{X}_{it}\theta$$

where y is the dependent variable (LCO_2_), X is the vector of explanatory variables (per capita income, square of per capita income, energy utilization, and urbanization). In this case, q is the same number of slacks across singular cross-sectional units *i* (number of countries in the panel = 7) in time *t* (experimental period, 1990 to 2015). In addition, Δ denotes the difference operator, ϕ is the adjustment coefficient, θ indicates the long-run coefficient that produces β and ψ adjudge the behavior of the model after reaching convergence while ε is the error term. In addition, this study adopts the causality investigation through Dumitrescu and Hurlin (2012). Considering the intention of providing a pairwise and directional panel relationship between the examined variables, the Granger causality approach of Dumitrescu and Hurlin (2012) is found effective in this context. Although the step-by-step approach is not provided in the case, inference from the estimated result is illustrated in the subsequent section.

## Results and discussion

The PMG panel analysis methodology within the framework of ARDL of Pesaran et al. (1999) provides the unique advantage of obtaining not just the long-run and short-run coefficients alone but also the cross-section short-run coefficients of individual countries. The results of both the long-run and short-run coefficients and the individual country cross-section outputs are provided in Tables 4 and 5 respectively.

**Table 4:** PMG ARDL results

*PMG Long-run Estimates*		
**Variables**	**Coefficients**	**P-value**
LogURB	-0.593672**	0.0334
LogEGC	0.827200****	0.0000
LogPCI	-0.067932**	0.0119
LogPCI^2^	0.006408***	0.0018
*Short-run Estimates*		
ECT	-0.611379***	0.0006
DLogURB	-2.919964	0.8319
DLogEGC	0.307027	0.6830
DLogPCI	-0.485224	0.4802
DLogPCI2	0.086380	0.6750
C	-0.526711***	0.0002

The subscripts *** and ** present the significance of estimates at 1% and 5% levels respectively. LogCO_2_ is carbon dioxide emissions, LogEGC is the energy use in kilograms of oil equivalent per capita, and LogURB is the proportion of the population living in the urban settlements. LogPCI and LogPCI2 are the real GDP per capita and the square values of the real GDP per capita respectively. The employed model is ARDL (1, 1, 1, 1, 1)

**Table 5:** Individual Country Cross Section results

Countries	Coefficients	P values
Algeria	ECT = -0.518368***	0.0005
	LogURB = 6.7707	0.9630
	LogEGC = -0.3272*	0.0818
	LogPCI = 0.5279	0.1192
	LogPCI2 = -0.1139***	0.0079
Angola	ECT = -1.297543***	0.0000
	LogURB = -78.8273	0.6085
	LogEGC = -2.6155*	0.0904
	LogPCI = -0.0936***	0.0017
	LogPCI2 = -0.0011***	0.0003
Egypt	ECT = -1.112757***	0.0000
	LogURB = 34.3289	0.6110
	LogEGC = -0.6086***	0.0006
	LogPCI = -3.7073	0.2290
	LogPCI2 = 0.9766***	0.0082
Libya	ECT = -0.425087***	0.0013
	LogURB = -6.7158	0.9754
	LogEGC = 0.5358***	0.0008
	LogPCI = 0.1085	0.7826
	LogPCI2 = -0.0154	0.6351
Morocco	ECT = -0.021708**	0.0111
	LogURB = 0.2553	0.9219
	LogEGC = 0.7228***	0.0001
	LogPCI = 2.0793	0.9310
	LogPCI2 = -0.8243	0.8089
Nigeria	ECT = -0.237748***	0.0019
	LogURB = 1.6024	0.9964
	LogEGC = 3.9969	0.1001
	LogPCI = -0.7413	0.5348
	LogPCI2 = 0.2164**	0.0212
South Africa	ECT = -0.666440***	0.0005
	LogURB = 22.1461	0.9481
	LogEGC = 0.4450***	0.0050
	LogPCI = -1.5701	0.5643
	LogPCI2 = 0.3663**	0.0442

The subscripts ***, **, and * presents the significance of estimates at 1%, 5%, and 10% levels respectively

Based on the long-run estimates in Table 4, both urbanization and energy use significantly affect the CO_2_ emissions in the panel of the seven countries. As expected, energy use increases the rate of emission among the countries as a 1% rise in its level expectedly increases CO_2_ emissions by approximately 0.83%. This finding correlates with several results from some contemporary studies on the positive impacts of energy consumption on carbon emissions (Alola et al. 2019a; Saint Akadiri et al. 2019; Alola et al. 2021; Alola and Joshua 2020). On the other hand, urbanization appears to have a significant impact on the level of carbon emission among the countries going by the magnitude of its coefficient. The coefficient shows that the effect of urbanization on carbon emissions in the panel of the examined countries is desirable because the direction of impact shows that the rate of urbanization improves environmental quality. It is worth noting that there is no consensus on the impact of urbanization on carbon emission in the literature as most studies are largely divided between the desirable and undesirable effects of urbanization on the environment (Shahbaz et al. 2014; Al-Mulali and Ozturk 2015; Al-Mulali et al. 2015; Alola et al. 2020; Asongu et al. 2020). For instance, Yao et al., (2018) noted that urbanization can have an abating effect on carbon emission but such effect could be reduced depending on how deep the level of urbanization is. The peculiarities of these countries vis-à-vis the structure of the economies and energy consumption pattern could be a major reason for this nexus. Some of the countries are currently experiencing a gradual rise in the use of renewable energy, especially hydro and solar energy through the attraction of various projects as foreign direct investments and technological transfer from abroad. However, most of such initiatives are often domiciled in the urban centers with little or no penetration to the deep rural areas where there is acute dependence on dirty energy sources for day-to-day economic activities.

Concerning the findings of income and square of income on environmental quality, the coefficient of the former came out negative while the latter was positive, thus invalidating the EKC hypothesis. For the EKC to hold in the countries under our analysis, it is expected that the coefficients for the LogPCI and LogPCI^2^ should be positive and negative respectively such that the relationship between income and carbon emission follows an inverted U-shape curve. The implication, in this case, is that increase in the individual income of the people is a good catalyst for improving environmental quality in the estimated panel countries, especially in the long run. However, the result shows that when income per capita is doubled, the environmental quality begins to deteriorate, thus leaving the government of the examined panel countries with the responsibility of combating the trade-off between improving the income level and environmental quality. These results resonate with the stance of Djellouli et al. (2022) that the EKC does not hold in the African context. The error correction term (ECT) follows the expected negative sign, and it is significant in the model showing that the speed of adjustment to equilibrium is at about 61.13% annually.

Furthermore, only in Angola is the U-shaped relationship between carbon emissions and income validated (see Table 5). In the other examined six countries, the EKC hypothesis is neither validated nor the U-shaped relationship outrightly verified. Additionally, individual country cross-section results reveal that adjustment to equilibrium is fastest in the cases of Angola and Egypt and slowest in the cases of Nigeria and Morocco. Finally, a panel causality test was conducted using the Pairwise Dumitrescu Hurlin Panel Causality on the direction of causality between our variables, and the output is provided in Table 6.

**Table 6:** Panel Causality

	W-Stat/Zbar-Stat					
Variables	LogCO_2_	LogURB	LogEGC	LogPCI	LogPCI^2^	CONCLUSION
LogCO_2_	_	5.4120/3.3409 ***	3.9829/1.8379	7.7914/5.8433 ***	6.9786/4.9885 ***	${LogCO}_{2}\to LogURB,LogPCI,Log{PCI}^{2}$
LogURB	5.5591/3.4955 ***	_	5.5850/3.5228***	9.9176/8.0793 ***	9.4899/7.6296 ***	$LogURB\to {LogCO}_{2},LogEGC,LogPCI,Log{PCI}^{2}$
LogEGC	1.6949/-0.5683	5.4061/3.3347***	_	6.7235/4.7202 ***	7.0061/5.0174 ***	$LogEGC\to LogURB,LogPCI,Log{PCI}^{2}$
LogPCI	1.8010–0.4566	5.1672/3.0834***	3.1317/0.9427	_	3.5871/1.4217	$LogPCI\to LogURB$
LogPCI^2^	1.6048–0.6630	5.3974/3.3256***	2.8545/0.6512	3.2577/1.0753	_	$Log{PCI}^{2}\to LogURB$

The superscripts ***, **, and * represent the rejection of null of no causality at 1%, 5%, and 10% levels of significance respectively

The causality results show that only urbanization among other variables granger causes CO_2_ emission among the countries. Besides, there is a bi-directional causality between urbanization and the level of income, energy use, and the amount of carbon emission from the countries in the study. With regards to energy use and income level, a uni-directional causality was obtained showing that causality runs from energy use to income level for the panel of countries in the study.

## Conclusion and policy recommendations

The nexus between carbon emission, energy use, and income level has been explored in this study in the case of the leading oil-producing African states given the level of urbanization being witnessed on the continent in recent times. The Pooled Mean Group (PMG) panel ARDL estimator was applied for the empirical analysis to explore the long-run and short-run dynamics of the relationship between the variables for the panel of selected African countries namely, Libya, Morocco, Nigeria, Algeria, Angola, Egypt, and South Africa.

The results confirm that the rising level of energy use significantly exacerbates the level of carbon emission among the countries in the study while growing urbanization significantly creates a negative impact on CO_2_ emission for the study. This result was further buttressed by the Granger causality test which reveals that urbanization strongly granger causes CO_2_ for the panel of the countries in the study. Additionally, the EKC hypothesis does not hold for the panel of countries in the study.

Therefore, based on these findings, we thereby recommend that these countries should effectively maximize the possible pollution-abating effects of urbanization among these countries. This can be achieved through effective urban planning to ensure that environmental pollution is being mitigated in the wake of deepening urbanization among the countries. Strategies must be put in place to ensure the growth of sustainable cities and environmentally friendly urban communities in these countries. Specifically, the authorities of each state need to provide adequate funding for greener urban mass transportation to reduce conventional energy-driven transport arrangements that currently dominate the means of urban commuting among these states. Through this action, any potential urban growth-related emissions can be drastically controlled thereby positioning these countries to continue to take advantage of the obtained desirable nexus between urbanization and carbon emission as seen in the analysis. Overall, the understudied African state in this context will be able to make more progress in achieving sustainable development goals (SDG-11).

Furthermore, considering the pollution-aggravating effects of energy utilization among these states, we also recommend the provision of adequate support and incentives for initiatives in renewable power generation. The massive advantages in renewables, especially hydro and solar potential among these nations should be tapped into. The authorities are encouraged to adequately support and finance large power projects in these forms of energy to cut down reliance on fossil energy consumption. Additionally, the authorities are encouraged to sponsor more investments in clean energy technologies and make related research and development (R&D) funding available. Doing this would go a long way in helping these nations to address the observed detrimental pollution effects of energy utilization and ultimately assist them in creating better ecologically sustainable energy portfolios towards attaining SDG-7.

Lastly, since our result confirms a uni-directional causality from energy use to income level for the countries, energy-related policies must be carefully designed to ensure that the right balance is attained between growth-enhancing policies and the energy conservation policy vis-à-vis carbon emission mitigation policy. As such, we further advise that the policymakers and stakeholders in the understudied African states should be well-guided and deliberate in taking proper caution to ensure a simultaneous balance between emission mitigation actions and the need to achieve sustainable economic growth as enshrined in SDG-8.

## Data Availability

Not Applicable.
